# Graduate entry and undergraduate medical students’ study approaches, stress levels and ways of coping: a five year longitudinal study

**DOI:** 10.1186/s12909-015-0284-7

**Published:** 2015-01-24

**Authors:** Sally Sandover, Diana Jonas-Dwyer, Timothy Marr

**Affiliations:** 1 The University of Western Australia, 35 Stirling Highway, Crawley, WA Australia; 2 Fremantle Hospital, Alma Street, Fremantle, WA Australia

## Abstract

**Background:**

Incorporating graduate students into undergraduate medical degree programs is a commonly accepted practice. However, it has only recently been recognized that these two types of students cope with their studies in various ways. The aim was to compare the learning approaches, stress levels and ways of coping of undergraduate (UG) and graduate entry medical students (GEMP) throughout their medical course.

**Methods:**

From 2007–2011 each of the five year cohorts of undergraduate and GEMP students completed four components of the study. The components included demographics, The Biggs’ R-SPQ-2 F questionnaire which determines students’ approaches to learning, the Perceived Stress Scale (PSS) used to rate students perceived stress during the past four weeks, and the Ways of Coping (WOC) questionnaire used to assess students’ methods of coping with everyday problems.

**Results:**

There was a consistent difference between UG and GEMP students approaches to learning over the five years. GEMP students preferred a deep approach while the UG students preferred a superficial approach to learning. This difference became more obvious in the clinical years. There was no statistically significant difference between the groups in stress levels. There were consistent differences in the ways the two groups coped with stress.

**Conclusions:**

There were significant differences in approaches to learning and ways of coping with stress between the UG and the GEMP students. These need to be considered when introducing curriculum change, in particular, redesigning an UG program for post graduate delivery.

## Background

The focus of this study developed out of the inclusion of graduate students from a wide variety of disciplines into the undergraduate (UG) medical course at The University of Western Australia (UWA). The combining of graduate students into an undergraduate medical program raised a number of issues concerning the various ways students can learn and cope with the stresses of study. A review of the literature on medical students’ learning approaches, stress levels, and ways of coping revealed that measuring these processes is complex and can be influenced by many factors. Each of these areas is presented.

Learning approaches are not fixed traits; rather, students adjust their approach depending on the learning environment and the assessment tasks provided. According to Entwistle and Peterson the context of the learning setting affects student learning because they try to identify what is expected of them according to their past experiences as well as through the current social setting [1].

Entwistle, believes that when students take a deep approach to their learning they relate ideas and look for patterns and principles to make meaning from what they are learning [2]. Pask also identified this as adopting a *holist* approach [3]. Another aspect of the deep approach to learning is to use evidence and examine the logic of an argument which, according to Pask, is known, as adopting a *serialist* approach [3]. Entwistle, McCune and Walker further say that the deep approach involves monitoring the development of the individual’s understanding [4]. In contrast, when students adopt a surface approach, unrelated bits of information are memorised leading to more limited learning [2]. There are many reasons why the deep learning approach should be encouraged. Research has shown that students who adopt a deep learning approach perform better in coursework and project work, are more satisfied with their course and do better in exams [5-10]. McManus, Richards, Winder and Sproston, reported that medical students adopting a deep approach had a better clinical experience than those who did not [11].

Many instruments have been developed to measure student learning approaches and in 2004 Coffield et al. reviewed 13 of the most influential models [12]. In higher education, one of the most well-known and commonly used is The Biggs’ R-SPQ-2 F questionnaire [13-16]. In general learning approach researchers agree that students can adopt either a deep approach (DA) to their learning where the intention is to understand, or a surface approach (SA) to their learning, which is goal oriented. In 2010, Baeten et al. reviewed over 93 studies identifying factors that encouraged or discouraged deep learning [17]. As a result they classified these factors into three areas: student, contextual, and perceived contextual factors [17]. These factors have been investigated as potential areas for changing students’ approaches to learning.

Medical students have been found to have high levels of stress throughout their medical training [18-22]. The main factors identified as contributing to stress levels were heavy workloads and coping with academic studies [21,23]. According to Stewart et al. and LeBlanc stress was also associated with poor academic performance [23,24].

Also of interest is the research that identified differences between stressors for undergraduate and graduate students. Factors, such as, family commitments, financial stress, lack of leisure time and social isolation have been reported to have the capacity to impact on graduate students more than undergraduate students [25-27]. In Rolfe et al.’s study the main stressor for undergraduate students compared with their graduate counterparts was the initial decision to study medicine [27].

Medical students adopt a variety of strategies to cope with stress [28,29]. The technique used to cope with a stressful situation is known as a coping strategy [30]. There are several ways to classify strategies for coping with stress. Folkman and Lazarus describe eight coping processes: Confrontative coping, Distancing, Self-controlling, Seeking social support, Accepting responsibility, Escape avoidance, Planful problem solving and Positive reappraisal (see Instruments,Ways of Coping (WOC) for further detail) [31]. An et al. used similar classifications, further grouping these into three categories of coping; active-cognitive; active-behavioural; and avoidant [28]. Despite the classification systems, researchers seem to agree that students can adopt several different processes to help them deal with the stresses of life and this has also been shown to be the case with medical students [18,29].

There appears however, to be little literature in general comparing the coping processes used by graduate and undergraduate students in tertiary education. Nevertheless, several researchers have suggested that medical educators should consider including both formal and informal sessions on ways of coping with stress for medical students [19,27,28,32].

For example, Compton et al. suggest that support be provided at specific transition times, such as the move from pre-clinical to clinical studies where stress levels are reported to increase [19].

**Figure 1:**
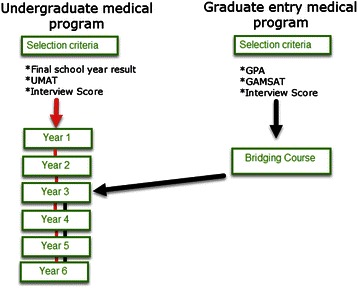
**Flow chart showing the entry pathways into the UWA undergraduate medical program.**

The UG medical course at UWA is a six year course with direct entry from school via the selection process which includes the results of their final school year exam, Undergraduate Medicine and Health Sciences Admission Test (UMAT) and interview score. The majority of students are 17 or 18 years of age, upon entry. To diversify the student intake and to meet increased demand for doctors, the Faculty of Medicine, Dentistry and Health Sciences introduced an alternative pathway into the UG medical program for mature aged students. The Graduate Entry Medical Program (GEMP) recognises prior learning by allowing graduate students who have completed a previous degree and who have met the entrance criteria (The Graduate Australian Medical Schools Admission Test (GAMSAT), interview process and Grade Point Average (GPA)) to enter the current UWA medical course in year three, instead of year one (Figure 1).

The GEMP students complete a compulsory intensive bridging course, consisting of an abridged year 1–2 UG medical curriculum. The course integrates material from nine disciplines and three Faculties over a 26 week period. The bridging process recognises the generic abilities graduates have gained through their varied education and work experiences, together with the assumed level of competencies assessed in the GAMSAT. GEMP students in this study entered the bridging course in 2007.

The UG medical course has been delivered at UWA for over 50 years. The introduction of GEMP students saw a significant change in student demographics in the medical course. The GEMP students are diverse in age, experience, prior study, occupation and financial status, and family responsibilities. As a result of this diversity, it was important to identify whether the learning environment in the medical program should have a corresponding change. This is imperative in light of the proposed introduction of new course structures at UWA, where all professional degrees, including medicine will be delivered to postgraduate students.

The results will be of value in determining whether any changes should be made to teaching styles, support services and selection processes in the new course structure.

## Methods

### Study design

Ethical approval for this research was given annually by the HREC at The University of Western Australia. Anonymous, annual, voluntary questionnaires were obtained from consenting students over 5 years from 2007 – 2011. This included a baseline questionnaire at the beginning of 2007. The results were categorised into two groups, UG and GEMP for the purposes of a pre-test/post-test cross-sectional analysis. With only 5 annual data points there was insufficient data to perform a longitudinal analysis of individual students through time. Instead the students were compared by group.

### Sampling and recruitment

Annual (including baseline) questionnaire data for the first two years (baseline, 2007, 2008) was obtained via voluntary paper based questionnaires disseminated after a lecture in the second term of each year. In subsequent years (2009–2011) surveys were voluntarily obtained electronically in term two, via online questionnaires.

### Participants

Participation was voluntary and anonymous. The participants were UG year 2 and GEMP medical students studying medicine in 2007. They completed each of the four components of the survey. At the beginning of the teaching period in 2007 each group completed a baseline survey. The student groups also completed the same survey annually for the duration of the degree course, ending in 2011.

### Instruments

A single questionnaire consisting of four parts was used to gather students’ demographic information, learning approaches, perceived stress levels and ways of coping.

Part 1, demographics included questions about age group, gender, previous study, year of study, work information and living arrangements. Part 2, was the Biggs’ R-SPQ-2 F questionnaire [13]. Part 3, was the Perceived Stress Scale (PSS) questionnaire and Part 4, consisted of The Ways of Coping questionnaire [31,33]. Parts 2–4 are now further described.

The Biggs’ R-SPQ-2 F questionnaire determines students’ approaches to learning as either, a deep approach (DA) an intention to understand, made up of two subscales, deep motive (DM) intrinsic interest and deep strategy (DS) maximise meaning or a surface approach (SA) fragmented or rote learning, made up of two subscales, surface motive (SM) fear of failure and surface strategy (SS) narrow target and rote learning [13].

The questionnaire consists of 20 items. Students respond to each item using a 5 point Likert scale (A = 1 this item is never or only rarely true of me, B = 2 this item is sometimes true of me, C = 3 this item is true of me about half the time, D = 4 this item is frequently true of me, E = 5 this item is always or almost always true of me). To obtain the main scale scores (DA & SA) 10 specific items are totalled for each. To obtain the sub-scale scores (DM, DS, SM & SS) 5 specific items are totalled for each [13]. The questionnaire has been used extensively in higher education. A commonly reported measure of a scale’s internal consistency is Cronbach’s alpha. An alpha of above 0.70 or above is considered to be acceptable [34].

The Perceived Stress Scale asks students to rate the stress perceived to be evident in their lives during the past four weeks [33]. It consists of 10 questions that ask how often they felt or thought a certain way. The students answer using a 5 point Likert scale (0 Never, 1 Almost Never, 2 Sometimes, 3 Fairly Often, 4 Very Often).

The Ways of Coping questionnaire is a theoretically derived measure with 66 Likert scale questions that can be used to assess students’ methods of coping with problems in their daily lives [31]. It measures individual’s coping processes, rather than coping dispositions or styles. Folkman and Lazarus recommend its use as a research tool in clinical settings [31].

The eight scales of the WOC questionnaire include:

1. Confrontative coping, where aggressive efforts are taken to alter the situation and suggests some degree of hostility and risk-taking;
2. Distancing, where cognitive efforts are made to detach oneself and to minimize the significance of the situation;
3. Self-controlling, where efforts are taken to regulate one’s feelings and actions;
4. Seeking social support when efforts are made to seek informational support, tangible support, and emotional support;
5. Accepting responsibility, where acknowledgement of one’s own role in the problem occurs with a concomitant theme of trying to put things right;
6. Escape avoidance, where wishful thinking and behavioural efforts are made to escape or avoid the problem. Items on this scale contrast with those on the Distancing scale, which suggest detachment;
7. Planful problem solving, where deliberate problem-focused efforts are made to alter the situation, coupled with an analytic approach to solving the problem; and
8. Positive reappraisal, where efforts are made to create positive meaning by focusing on personal growth. It also has a religious dimension.

### Data analysis

Baseline and annual survey data were analysed by comparing the groups’ data as a whole (pooled data) and by comparing the results by year (2007–2011). These two groups of students (GEMP and UG) were treated as independent populations, rather than co-dependent. With the exception of patient age, the questionnaire information was classified as ordinal data and the relevant statistical tests used for analyses were the Median and the Kruskal-Wallis tests, which can be used to derive inference about differences in median and variance values of ordinal data respectively. The appropriate graphical technique is the box plot. For accurate statistical reporting of non-parametric tests, we have included both the Median Test to analyse whether two samples are equivalent in terms if central tendency (equivalent is Mean in parametric tests) and standard deviation/variance (ie Kruskal Wallis Test), to analyse whether two populations originate from the same distribution or whether they are truly independent samples. Information from the surveys was entered into Microsoft Excel and IBM Statistical Package for the Social Sciences (IBM SPSS 19) for analysis.

## Results

### Baseline survey

One hundred and thirty two students participated in the baseline survey. Of these, 76 (63% response rate) were second year UG and 56 (95% response rate) were GEMP students.

### Annual survey

In total over five years, five-hundred and seventy nine observations were obtained constituting the pooled data. This comprised 392 (43% response rate) UG student observations and 187 (64% response rate) GEMP student observations. On average, 78 (57% response rate) UG and 37 (63% response rate) GEMP students responded each year (Table 1).

**Table 1:** **Descriptive statistics for annual and baseline survey (gender, age group and year)**

	Baseline		2007		2008		2009		2010		2011		TOTAL	
	UG	GEMP	UG	GEMP	UG	GEMP	UG	GEMP	UG	GEMP	UG	GEMP	UG	GEMP
N	76	56	60	48	108	48	96	50	61	19	67	22	392	187
Gender														
Male	26	20	22	15	44	19	36	19	19	5	24	8	145	66
Female	50	36	38	33	64	29	60	31	42	14	43	14	247	121
Age range														
18 - 22	73	22	56	15	99	6	69	1	37	0	10	0	271	22
23 - 25	2	21	3	18	7	25	21	24	21	6	47	2	99	75
26 - 30	1	9	0	11	1	10	4	15	3	5	10	12	18	53
31 - 50	0	4	1	4	1	7	2	10	0	8	0	8	4	37

### Demographics

#### Learning approaches

Undergraduate and GEMP students were compared in their approaches to learning. At baseline, statistically significant differences in approaches to learning existed between the two groups in both the Median and Kruskal-Wallis Tests (Table 2). GEMP students showed a preference for all levels of deep learning and UG students showed a preference for all levels of superficial learning. Pooled data showed similar results.

**Table 2:** **Median and Kruskal-Wallis Test Results for Approaches to Learning by Group**

*Baseline*							
*Median Test*					*Kruskal-WallisTest*		
					*Mean Rank*		
Approach to Learning		UG	GEMP	*p-value*	UG	*GEMP*	*p-value*
Deep Approach	> Median	29	35	0.006*	57.41	78.84	0.001*
	<= Median	47	21				
Deep Motive	> Median	30	28	*0.228*	59.01	76.67	0.008*
	<= Median	46	28				
Deep Strategic	> Median	26	33	0.005*	57.52	78.69	0.002*
	<= Median	50	23				
Superficial Approach	> Median	47	18	0.001*	77.31	51.83	0.000*
	<= Median	29	38				
Superficial Motive	> Median	40	14	0.001*	77.51	51.55	0.000*
	<= Median	36	42				
Superficial Strategic	> Median	45	20	0.008*	75.50	54.29	0.002*
	<= Median	31	36				
***Pooled Annual***							
***Median Test***					***Kruskal-WallisTest***		
					***Mean Rank***		
**Approach to Learning**		**UG**	**GEMP**	***p-value***	**UG**	**GEMP**	***p-value***
Deep Approach	> Median	181	101	0.078**	274.84	321.78	0.002*
	<= Median	211	86				
Deep Motive	> Median	147	94	0.004*	270.11	331.7	0.000*
	<= Median	245	93				
Deep Strategic	> Median	146	72	*0.770*	281.6	307.6	0.079**
	<= Median	246	115				
Superficial Approach	> Median	205	66	0.000*	309.73	248.64	0.000*
	<= Median	187	121				
Superficial Motive	> Median	199	85	*0.232*	307.86	252.55	0.000*
	<= Median	193	102				
Superficial Strategic	> Median	188	60	0.000*	308.61	250.99	0.000*
	<= Median	204	127				

*Significant with 95% confidence.

**Significant with 90% confidence.

**Figure 2:**
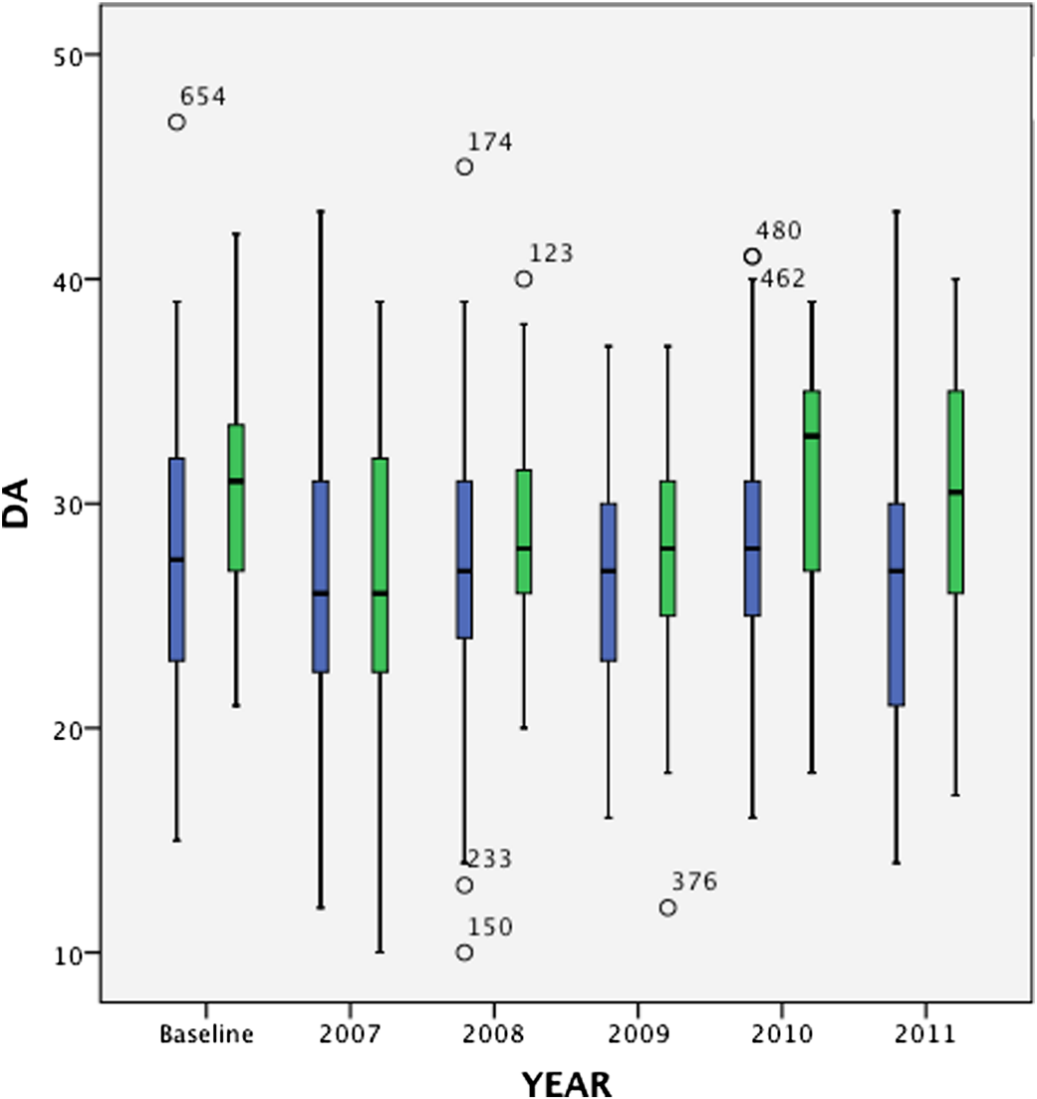
**Deep Approach to Learning by group over the five years of the study (Y2-Y6 of UG medical program).** Legend text UG = Blue GEMP = Green.

**Figure 3:**
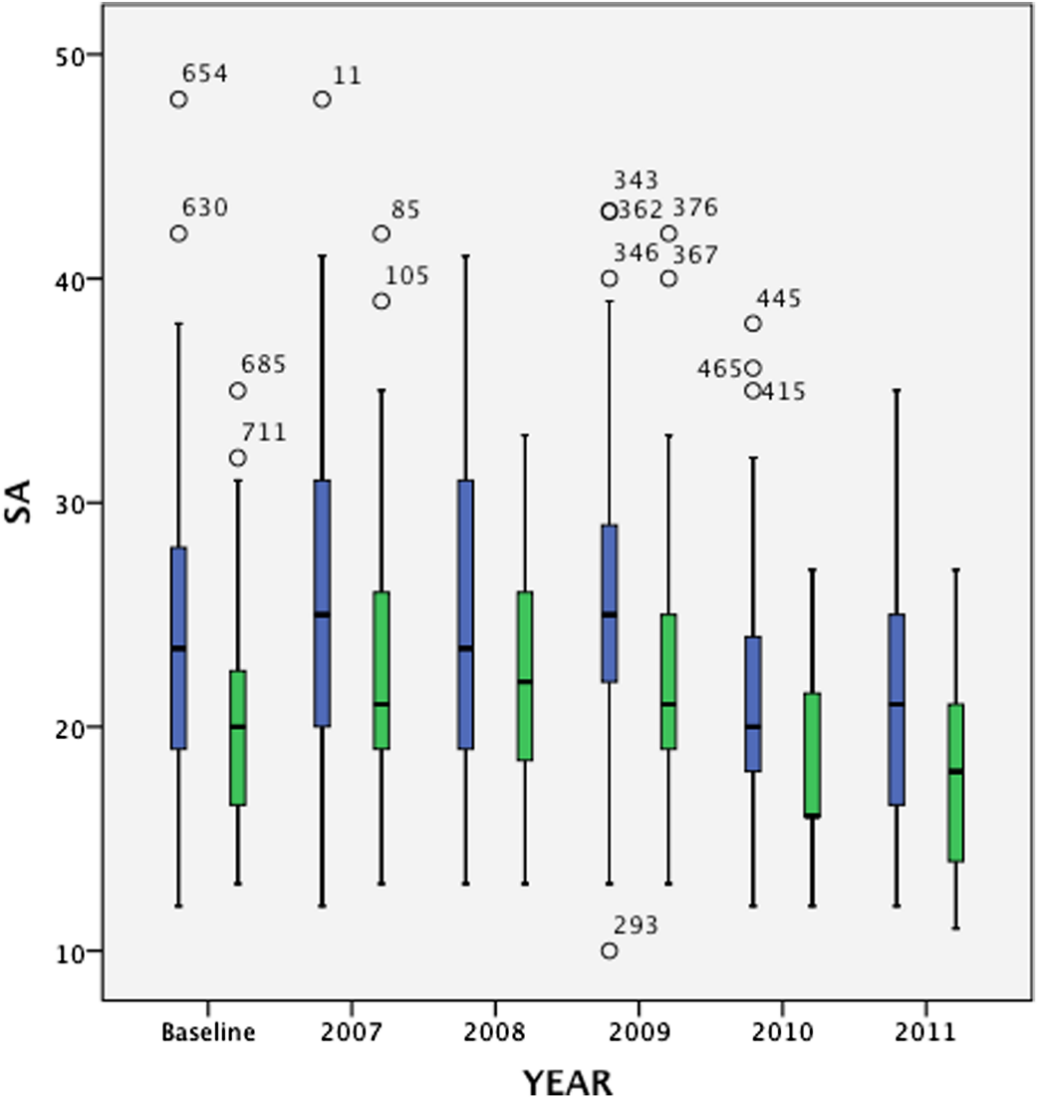
**Surface Approach to Learning by group over the five years of the study (Y2-Y6 of UG medical program).** Legend text UG = Blue GEMP = Green.

An analysis of the comparison of each learning approach between the groups (annual data) over the five years showed variations between the groups (Figures 2 and 3). The results support the pooled sample findings that a consistent difference exists between UG and GEMP students for deep motive and superficial approaches to learning. At baseline Kruskal-Wallis tests for DA and SA between the two groups were significantly different (DA *p = 0.001* SA *p = 0.001*). These differences persisted throughout the five years of the study (DA *p = 0.02* SA *p = 0.01*). The difference in DA that existed at baseline was reduced at the end of the bridging course (2007) and increased in the last two years of the course (2010–2011) shown in Figure 2. The difference in SA was also statistically significant at baseline and remained so, with the exception of 2008 (year 3 of the course) throughout as shown in Figure 3. Chronbach’s alpha was calculated for each of the identified approaches, each year. The alpha was above 0.70 in both items, each year. For DA it ranged from 0.75 to 0.86 and for SA it ranged from 0.81 to 0.86.

### Deep and superficial approaches

The progressive divergence between groups from 2007–2011 in the use of a deep approach to learning is evident (Figure 2) with GEMP students increasingly adopting this style of learning relative to UG students. The greatest divergence occurs in the clinical years of the medical program.

In contrast, the preference for surface approaches to learning decreased over the five years in both groups. However, there is a marked difference between the two groups with GEMP students having a significantly lower preference for surface learning compared with UG students.

### Perceived stress

Perceived stress scores were measured and compared each year. There was a difference between the UG and GEMP groups at baseline (p = 0.000) but, no statistically significant difference between the UG and GEMP groups in any of the subsequent years. UG students recorded higher stress levels at baseline.

### Ways of coping

The baseline and pooled data analysed by Median Test and Kruskal Wallis are displayed in Table 3.

**Table 3:** **Ways of Coping, Median Test and Kruskal-Wallis Test - a comparison of coping strategies by group**

*Baseline*							
	*Median Test*				*Kruskal-Wallis Test*		
					*Mean Rank*		
Coping Strategy		UG	GEMP	*p-value*	*UG*	*GEMP*	*p-value*
Confrontive Coping	> Median	85	15	0.006*	121.96	93.5	0.005*
	<= Median	88	41				
Distancing	> Median	81	18	0.076**	119.91	99.83	0.048*
	<= Median	92	38				
Self Controlling	> Median	79	22	0.496	118.63	103.79	*0.144*
	<= Median	94	34				
Seeks Social Supports	> Median	78	25	0.923	113.46	119.76	*0.535*
	<= Median	95	31				
Accepting Responsibility	> Median	96	14	0.00*	125.77	81.73	0.000*
	<= Median	77	42				
Escape Avoidance	> Median	90	17	0.008*	124.03	87.12	0.000*
	<= Median	83	39				
Planful Problem Solving	> Median	59	31	0.008*	107.51	138.13	0.037*
	<= Median	114	25				
Positive Reappraisal	> Median	81	24	0.717	115.6	113.12	*0.808*
	<= Median	92	32				
***Pooled Annual***							
	***Median Test***				***Kruskal-Wallis Test***		
					***Mean Rank***		
**Coping Strategy**		**UG**	**GEMP**	***p-value***	**UG**	**GEMP**	***p-value***
Confrontive Coping	> Median	212	74	0.001*	306.95	254.46	0.000*
	<= Median	180	113				
Distancing	> Median	202	67	0.000*	310.09	247.88	0.000*
	<= Median	190	120				
Self Controlling	> Median	173	75	*0.360*	299.83	269.39	0.040*
	<= Median	219	112				
Seeks Social Supports	> Median	165	92	*0.108*	285.35	299.75	*0.331*
	<= Median	227	95				
Accepting Responsibility	> Median	208	77	0.007*	302.45	263.91	0.009*
	<= Median	184	110				
Escape Avoidance	> Median	198	67	0.001*	309.08	249.99	0.000*
	<= Median	194	120				
Planful Problem Solving	> Median	154	92	0.024*	277.38	316.45	0.008*
	<= Median	238	95				
Positive Reappraisal	> Median	192	88	*0.665*	291.85	286.12	*0.699*
	<= Median	200	99				

*Significant with 95% confidence.

**Significant with 90% confidence.

The results indicate that UG students favour ‘confrontive coping’, ‘distancing’, ‘accepting responsibility’ and ‘escape avoidance’ as strategies compared with GEMP students who favour ‘planful problem solving’.

The baseline data showed a significant difference between the genders with females ‘seeking social support’ (*p = 0.002*). An analysis of differences in median values by gender for pooled data, indicates that males prefer ‘self controlling’ (*p = 0.017*) as a coping strategy relative to females. In contrast, females associated more strongly with ‘seeking social support’ (*p = 0.000*) and planful problem solving (*p = 0.047*) compared with males.

## Discussion

The UG and GEMP groups were markedly different at commencement and throughout this study. The two groups varied in all the areas surveyed; their approaches to learning, perceived stress levels and ways of coping.

The students were distinct in their approaches to learning. Overall, the GEMP students had a preference for deep learning while the UG students had a preference for surface learning. The discussion of factors that impact on approaches to learning in the current study is organised around the three factors: student; contextual; and perceived contextual factors as identified by Baeten et al. [17].

The difference in approaches to learning is not surprising, as age has previously been shown to be a factor that can impact on learning [17,35,36]. The five years of pooled data in the current study clearly identified the strong preference GEMP students had for deep approaches and the preference that UG students had for surface approaches to learning. One explanation for this could be the difference in ages between GEMP and UG students.

Baeten et al. also identified other *student factors* that have been shown to promote deep learning. These include; previous work experience, academic learning and skills, educational experiences, coping styles, self-direction in learning, learning habits and preference for teaching methods, motivation and enjoyment in learning [17]. Some of these factors are also clearly related to age and may have an influence on approaches to learning.

When examined year by year, over the five years, the size of the difference in approaches to learning between the GEMP and UG students varied. Differences in the learning environment over the five years, including teacher approaches, assessment, feedback and interactive group-work may have been responsible for this variation [17,36,37]. These factors have been identified by Baeten et al. as *contextual factors* [17].

Clarity of goals, freedom in learning, fragmented knowledge/relevance and perceived quality of teaching are factors that Baeten et al. [17] identified as *perceived contextual factors* which similarly impact on approaches to learning. Perception of excessive or inappropriate workloads is another element that has often been reported as influencing the adoption of surface approaches [38-40].

The impact of the contextual and perceived contextual factors should be viewed in light of the structure of the course at the UWA. The second and third year of the medical course at the UWA are considered to be pre-clinical years, with large group lectures and limited hospital exposure. The third year of the medical course is well known for its large volume of lecture based content and reproduction of content for exams. Fourth to sixth years are primarily conducted in small groups with clinical rotations, within the hospital and general practice settings.

Interestingly, the GEMP and UG students were not different in their approaches to learning in the third year of the medical course, with both groups exhibiting a reduction in preference for deep learning and associated increases in surface learning. This further supports the findings of other researchers in that the contextual and perceived contextual factors can influence students’ approaches to learning [17,36].

Also, of interest was whether the two groups of students’ preferences for learning approaches would converge as they progressed from the pre-clinical to the clinical stages of the medical program. This change was not seen in the current study. In fact, the preferences between the two groups diverged, with the GEMP students increasing their preference for deep learning in the final two years of the medical program compared with the UG students who maintained a preference for surface learning. The change in the learning environment from pre-clinical to clinical and associated assessments in year five and six appear to have had an impact on the GEMP students, without bringing about a corresponding change in the UG students.

Our results support the findings of Gijbels et al. and Baeten et al. who identified that the initial approach to learning at the moment of entering the learning environment is predictive of the approach students will adopt [17,36]. Further, they found that the stronger the initial approach, the less likely that the approach would change. Of importance, they found that students rarely changed their approach from a surface to a deep approach to learning [17,36]. However, they did find that students were more readily able to change from a deep to a surface approach to learning. Similarly, Marton and Saljo also found it was easier to induce surface learning, but found a change to deep learning to be extremely difficult [41]. McManus et al. reported that the learning approach adopted by medical students in the final year of study can predict the amount of knowledge gained from the clinical experience in their final year of study, with a deep approach predicting a greater gain [11]. McManus et al. also showed that this relationship was valid beyond the final year, with a greater clinical experience in the final year predicted from the students who adopted a deep approach at application to medical school [11].

Undergraduate students in the current study made very few changes over the five years from the relatively strong surface approach to learning at the commencement of the study. The GEMP students, however, reported significantly higher levels of deep learning approaches compared with the UG students at the commencement of their studies and changed to more surface learning when the learning environment dictated. Of importance however, was the capacity of these students to change to deep learning when the environment promoted this. The GEMP students reported significantly higher levels of deep learning approaches in the final years of the course. If a deep approach to learning is related to a better clinical experience, irrespective of length of clinical experience, as previously reported by McManus et al., it is clearly crucial that every effort must be made to shift UG students from their preference for a surface approach, to a deep approach to learning, to try to maximize the clinical experience [11]. How and when to do this remains the challenge.

The two groups reported different levels of perceived stress at the commencement of the study. The reduced stress levels of the GEMP students could perhaps be explained by the timing of the survey. The GEMP students had not commenced their medical education, and were extremely excited at being accepted into medical school. The second year UG students on the other hand were one year into their medical program. Both groups reported the same levels of perceived stress throughout the five years of the study. This was a surprise, as GEMP students appeared to have more external stressors than UG students. For example, caring and supporting family and children, hours of work in a previous profession, caring for aging parents and financial considerations. It is possible the results indicate the intricacies of ‘perceiving’ stress levels rather than actual stress. Again perceived stress is a student factor that impacts on approaches to learning.

Ways of coping with a high demand course also varied between the two groups. The variations were consistent throughout the five years. Undergraduate students coped by using confrontative and escape avoidance mechanisms. Graduates primarily preferred planful problem solving. Again, these preferences could be a function of age. What is important is that the differences persisted throughout the medical course. The relationship between stress, coping processes, and learning, appears to be complex. An et al. in their study identified that medical students using an avoidant coping processes experienced high levels of academic stress [28]. Stewart et al. and LeBlanc found that academic stress was related to poor academic performance [23,24]. Trigwell, Ellis and Han in recent research, identified a relationship between emotions and learning approaches, and academic performance [42]. They found that students were more likely to adopt a deep approach to learning if they experienced strong positive emotions such as hope, pride and confidence. Students were more likely to adopt a surface approach to learning if they experienced strong negative emotions such as anger, boredom, anxiety and shame. Higher academic performance was achieved with deep approaches and positive emotions. Baeten et al. includes emotions in the student category of factors that impact on learning [17]. Performance avoidance and fear of failure were related to surface learning, while self-confidence, and self-efficacy were related to deep approaches to learning [43,44].

Medical students are presented with many stressors throughout their medical studies. From the results of the current study, it appears that GEMP and UG students use different processes to deal with stressors. It is possible the processes used to cope with stresses are related to the approaches students take to learn. It is however, difficult to determine if there is a causal pathway. If entrance to medical school with deep approach to learning is predictive of a good clinical experience, it seems appropriate that this criterion is considered as an important part of the entrance selection process. Changing medical studies from undergraduate to post graduate studies may be a way to enhance this process.

In addition, the way students respond to stressors throughout the medical course appears to impact on their approaches to learning. The UG students primarily and consistently responded to stress with emotional responses that is, they view stressors as being out of their control and not able to be changed [31]. The GEMP students responded with problem solving processes. It is possible that the emotional processes preferred by the UG students in response to stress, could be classified as negative emotional responses which Trigwell et al. found to be predictive of a surface approach to learning [42]. Based on these results medical schools need to consider how to assist students with positive coping mechanisms for stress reduction throughout their course with the subsequent aim of encouraging deep learning strategies.

## Conclusions

In designing a course and promoting deep learning, *student factors* such as, the age of the student groups and characteristics such as, initial learning preferences, motivation, self-confidence and self-efficacy and coping processes should be considered. Though not able to be manipulated, these factors have an impact on student approaches to learning along with the more well-known contextual/learning environment factors that remain the domain of the course developer.

If educators are aware of the learning approach preferences that students have at the beginning of their courses then the learning environment can be developed to support a deep approach. In addition, as academic developers and teachers we should assist the students with the stressors of completing a medical degree and provide positive emotional strategies to help them cope. To ignore these factors will not do justice to optimal student learning.
